# Peer norm guesses and self-reported attitudes towards performance-related pay

**DOI:** 10.1371/journal.pone.0174724

**Published:** 2017-04-17

**Authors:** Nikolaos Georgantzis, Efi Vasileiou, Iordanis Kotzaivazoglou

**Affiliations:** 1 School of Agriculture Policy and Development, University of Reading, Reading, United Kingdom; 2 Laboratori d’Economia Experimental and Economics Department, Universitat Jaume I, Castellon, Spain; 3 University of Sheffield, International Faculty, City College, Thessaloniki, Greece; 4 Technological Educational Institute of Central Macedonia, Serres, Greece; Middlesex University, UNITED KINGDOM

## Abstract

Due to a variety of reasons, people see themselves differently from how they see others. This basic asymmetry has broad consequences. It leads people to judge themselves and their own behavior differently from how they judge others and others’ behavior. This research, first, studies the perceptions and attitudes of Greek Public Sector employees towards the introduction of Performance-Related Pay (PRP) systems trying to reveal whether there is a divergence between individual attitudes and guesses on peers’ attitudes. Secondly, it is investigated whether divergence between own self-reported and peer norm guesses could mediate the acceptance of the aforementioned implementation once job status has been controlled for. This study uses a unique questionnaire of 520 observations which was designed to address the questions outlined in the preceding lines. Our econometric results indicate that workers have heterogeneous attitudes and hold heterogeneous beliefs on others’ expectations regarding a successful implementation of PRP. Specifically, individual perceptions are less skeptical towards PRP than are beliefs on others’ attitudes. Additionally, we found that managers are significantly more optimistic than lower rank employees regarding the expected success of PRP systems in their jobs. However, they both expect their peers to be more negative than they themselves are.

## 1. Introduction

It has been often documented that people tend to overestimate the extent to which their beliefs coincide with a given social norm. Psychologists refer to this phenomenon with the term “false consensus”.[1]. From a statistical point of view, it looks rather straightforward that, in the absence of systematic biases, individual beliefs and individual perceptions of peer norms should coincide at least on average. This is also compatible with a broadly accepted principle according to which social norms play a central role in the formation of individual beliefs and behavior [2] favoring the emergence of a (real) social consensus and “modal conformism” of individual preferences. However, research in the field of social psychology has found that people often misperceive the norms in their peer group. According to Epley and Dunning [3], such differences in how people see themselves versus others are rooted in basic processes of human perception. It is almost axiomatic that as long as people are in a position to perceive themselves and to perceive others, differences in those perceptions will exist and will engender disagreement, misunderstanding, and conflict. Due to a variety of reasons, people see themselves differently from how they see others. They are immersed in their own sensations, emotions, and cognitions at the same time that their experience of others is dominated by what can be observed externally. This basic asymmetry has broad consequences. It leads people to judge themselves and their own behavior differently from how they judge others and others’ behavior [4]. Recent research in food choice [5] has revealed a systematic pattern of divergence between individual preferences and individual beliefs regarding peer norms. In particular, it has been observed that individuals tend to state a stronger (weaker) preference for healthy (less healthy) food than they believe their peers do. The divergence vanishes for food which is considered to be “neutral” (not particularly healthy or unhealthy). Therefore, individuals tend to overestimate their acceptance of a socially desirable rule compared to what they would guess regarding others. Humphrey [6] found that subordinate workers undervalue their fellows’ subordinates and overvalue their managers. His findings are based on the structural cognitive model assuming that organizational structures influence the information that actors have about each other. The magnitude of the difference in how you perceive yourself from how you perceive others is systematic and predictable. For example, “closer” others are perceived in more self-like ways than more “distant” others [7]. However, the sign of the difference and its determinants are not fully understood.

Our research also relates to the literature on extrinsic versus intrinsic incentives in labor relations. Following the literature on gift exchange, a reliable worker would reciprocate to a generous employer in a way which does not require the performance-contingent, ex ante establishment of compensating incentives rules like PR contracts [8]. In the Akerlof [8] model, higher pay always leads to higher effort (though firms clearly would not wish to pay for unbounded effort) and if worker effort depends on a reference wage, then it may be logical for firms to pay a wage above that level to obtain extra effort. However, extrinsic incentives may "crowd out" intrinsic ones, yielding the contrary effects to the pretended increased motivation and higher effort [9, 10]. Behavioral economists recognized that extrinsic incentives (rewards) may positively affect individuals only in the short run, but in the long run they might decrease motivation [11]. They explain that extrinsic motivation has a limited impact on actual performance while it may reduce the agent's motivation to undertake similar tasks in the future. In fact, [12] warn us that "there is no doubt that the benefits [piece-rate systems or pay-for-performance incentive devices] can be considerably compromised when the systems undermine workers' intrinsic motivation. Some of these findings are confirmed by the perceived potential effects of Performance-Related Pay (PRP) on intrinsic motivation. A variety of psychological theories have been used to designate the role of pay in motivation. Nevertheless, expert opinions for the effectiveness of pay as a motivator have historically been divided. Motivational theories can be classified in two groups, according to the importance they assign to pay as a motivator. The first group of theories, including Maslow’s need hierarchy theory [13], Herzberg’s motivation-hygiene theory [14] and Ryan and Deci’s [15] self-determination theory, questions the contribution of monetary rewards to performance enhancement. It mainly emphasizes internal motives and intrinsic rewards, such as recognition, social relations, self-actualization, job enrichment or autonomy. It asserts that money helps employees to meet only ‘basic needs’ and prevents dissatisfaction; however, pay cannot promote satisfaction. Hence, the motivational effect of financial incentives may be not only limited, but also negative [16]. On the contrary, the second group of theories, including reinforcement theory, expectancy theory, equity theory or goal-setting theory, mainly concentrates on the process by which desired behavior is stimulated and enhanced, and advocate that there is a strong, positive pay-motivation relationship [17]. In general terms, these theories argue that individuals are motivated when they are challenged and reinforced to attain clear, measurable and demanding but reasonable goals with valued and fair rewards. They imply that for most employees, money is the primary reason for working; therefore, pay is potentially a very effective means for improving performance [18].

We report results on the elicitation of beliefs and peer norm guesses providing evidence on the divergence between individual attitudes and guesses on peers’ attitudes. We also find evidence on the perceived negative effects of PRP on intrinsic motivation. Respondents report a more optimistic expectation regarding the success of a PRP scheme in their working environment as compared to their guesses on their peers’ expectations. We find that managers are more optimistic than clerical employees regarding the success of the scheme, but they both expect their peers to be more negative than they themselves are. Overall, we believe that understanding the existence of these misperceptions may help us to understand perceived norms and behavioral patterns in the working environment in order to successfully implement necessary reforms in the public sector.

## 2. Data and measures

### 2.1. Respondents and procedure

The current study is based on data from public servants in northern Greece. The questionnaire was administrated in January 2015 at the National Centre for Public Administration and Local Government (EKDDA) and more specifically its decentralized Thessaloniki annex, PINEPTH. PINEPTH covers the northern part of Greece and serves a population approaching 40% of the public servants in Greece. The Thessaloniki Section of the Institute of Life-Long Education (INEΠ) has among its objectives to educate those working in the public sector and the local administration. The Institute belongs to the National Centre for Public Administration and Local Government (EKDDA) which is the national strategic agent for the development of Human Resources of the Public Administration and Local Government (http://www.ekdd.gr/ekdda/index.php/en/2012-06-29-09-59-33). While attendance in the courses is voluntary, participation of public employees is very high because of the weight the courses have in promotions to a higher administrative rank. Therefore, access to the participants in the courses of the institute guarantees a representative sample, including people from a broad range of geographical areas, hierarchy ranks and institutions. While the INEΠ evaluates participants in the framework of the aforementioned courses and it may carry out its own research, the survey whose data are used hereby was administered independently with a clear indication that the information would be used under strict anonymity, exclusively for the purposes of this research, which was undertaken by autonomous researchers belonging to academic institutions only.

A permission was first guaranteed by the INEΠ for the survey to be distributed to the attendants in its courses. Further, it was made clear to them, that individual participation was voluntary. The Ethics Committee of the School of Agriculture Policy and Development at the University of Reading approved this study.

We distributed a total of 840 questionnaires during the first break of the morning class. Responses were collected at the end of the same day, allowing for about five hours within which the survey could be completed. A relatively high rate of responses was obtained, with 584 questionnaires returned to us fully or partially completed. To avoid the problem of missing variables, we used the sample of 520 complete responses.

The primary data for our assessment of PRP traits took the form of questionnaire responses indicating personal perceptions of, and attitudes towards, general practice in the context of the forthcoming adoption of PRP in the Greek public sector. It must be stressed that the definition of PRP incentives and their precise characteristics are still subject to debate. As no standard instrument for the assessment of PRP traits being available for this context, we designed our own, as follow.

Each respondent was confronted with 11 questions, on each of which he/she was invited to express an opinion on a 5-point scale. The scale ranged through strongly disagree (1), disagree (2), unsure (3), agree (4), and strongly agree (5). Table 1 reports the definition of each variable. The questions are numbered according to their appearance on the original questionnaire. Frequency distributions of responses to each of the 11 attitudinal statements appear in Table 2.

**Table 1:** Variables List.

Variables	Definition
***Attitudinal traits***	
Q 1. A PRP system help the workforce to improve its productivity	Dummy variable equal to 1 if the individuals; reported the highest score in the five-point scale and 0 otherwise
Q. 2. A PRP system help public servants to better understand the organization values and priorities	Dummy variable equal to 1 if the individuals; reported the highest score in the five-point scale and 0 otherwise
Q. 3.A PRP systems in public administration discourage low-skilled applicants	Dummy variable equal to 1 if the individuals; reported the highest score in the five-point scale and 0 otherwise
Q. 4.A PRP system prompt employees to be interested in tasks related to financial incentives	Dummy variable equal to 1 if the individuals; reported the highest score in the five-point scale and 0 otherwise
Q. 5.A PRP system lead a public servant to an unethical behavior	Dummy variable equal to 1 if the individuals; reported the highest score in the five-point scale and 0 otherwise
Q. 6.A PRP systems demotivate public servants that are intrinsically stimulated	Dummy variable equal to 1 if the individuals; reported the highest score in the five-point scale and 0 otherwise
Q. 7.A PRP system influence positively: supervisor-employee relationship	Dummy variable equal to 1 if the individuals; reported the highest score in the five-point scale and 0 otherwise
Q. 8.A PRP system influence positively: relationships with colleagues	Dummy variable equal to 1 if the individuals; reported the highest score in the five-point scale and 0 otherwise
Q. 9.A PRP system influence positively: total pay	Dummy variable equal to 1 if the individuals; reported the highest score in the five-point scale and 0 otherwise
Q. 10.A PRP system influence positively: sense of job security	Dummy variable equal to 1 if the individuals; reported the highest score in the five-point scale and 0 otherwise
Q. 11. A PRP system influence positively: tensions in work	Dummy variable equal to 1 if the individuals; reported the highest score in the five-point scale and 0 otherwise
***Control variables***	
Male	Dummy variable equal to 1 if the respondent is a male
Job satisfaction	Standardized score of satisfaction with the job or main activity where is measure on a seven-point scale of 1 = totally dissatisfied to 7 = totally satisfied
Clerk	Dummy variable equal to 1 if the respondent has a clerical position
***Dependent Variables***	
Own perception of the effective implementation of a PRP system	Standardized score of an individuals’ own perception where is measure on a seven-point scale of 1 = totally disagree to 7 = totally agree
Own perception of others’ perceptions of the effective implementation of a PRP system	Standardized score of an individuals’ own perception of others ‘perceptions where is measure on a seven-point scale of 1 = totally disagree to 7 = totally agree

**Table 2:** Questionnaire responses, Descriptive statistics.

Questions	Responses (%)				
**Independent variables—Attitudinal traits**	**Scale 1**	**Scale 2**	**Scale 3**	**Scale 4**	**Scale 5**
Q. 1. In your opinion, to what extend does a PRP system in public service help the workforce to improve its productivity?	7.4	9.5	16.6	47.8	18.7
Q. 2. In your opinion, to what extent does a PRP system help public servants to better understand the organization values and priorities?	11.7	18	19.4	41.7	9.2
Q. 3. In your opinion, to what extent do PRP systems in public administration discourage low-skilled employees (applicants)?	18.8	21.5	22.7	27.3	9.7
Q. 4. In your opinion, to what extent do PRP systems prompt public servants to be interested solely in tasks that are directly related to financial incentives?	2.7	6.5	20	51.3	19.5
Q. 5. In your opinion, to what extent may a PRP system lead a public servant to an unethical behavior?	12.6	20.4	24.6	32.2	10.2
Q. 6. In your opinion, to what extent may PRP systems demotivate public servants that are intrinsically stimulated?	21	21.9	25.2	24.4	7.5
*Scale from 1 (negative) to 5 (positive)*	**1**	**2**	**3**	**4**	**5**
Q. 7. In your opinion, in which way could an introduction of a PRP system influence: supervisor-employee relationship	12.8	20.2	34.7	21.9	10.4
Q. 8. In your opinion, in which way could an introduction of a PRP system influence: relationships with colleagues	21.5	31.3	31.5	11.3	4.4
Q. 9. In your opinion, in which way could an introduction of a PRP system influence: total pay	3.6	6.2	22.4	41.2	26.6
Q. 10 In your opinion, in which way could an introduction of a PRP system influence: sense of job security	17.8	19.8	33.3	20.6	8.5
Q. 11. In your opinion, in which way could an introduction of a PRP system influence: tensions in work	16	19.4	23.3	26.8	14.5
Your Job satisfaction from 1 to 7	11.1	13	22.5	31.4	22
**Dependent Variables**	**Strongly disagree (Scale 1–2)**	**DisagreeScale 3**	**UnsureScale 4**	**Agree Scale 5**	**Strongly agree**
					**(Scale 6–7)**
In your opinion, could an introduction of a PRP system be effectively implemented in your job (section)?	27.9	13.5	16.8	21.9	19.9
In your opinion, how could be your colleagues ‘reaction to the introduction of a PRP system?	39.8	19.1	18.9	13.8	8.4

The questionnaire additionally requested information on respondents´ on personal information (such as gender) and on Job characteristics (position in service and job satisfaction). The questionnaire included demographics and other individual information like years of employment in the public sector, education, age, but have been omitted from the model, as they did not appear to be significant in any of the specifications estimated.

### 2.2. Measures of “own perception” and “my perceptions on others’ perceptions”

As stated previously, people hold heterogeneous beliefs on others’ expectations. In order to consider whether there is divergence between first- and second-order beliefs (my beliefs on others’ beliefs). we use the following two questions allowing for “me versus others” comparisons. Therefore, the survey contained a question about employee’s own behavior (e.g. *“In your opinion*, *could an introduction of a PRP system be effectively implemented in your job (section)?”*), and a question about respondents’ perceptions of others’ attitudes (e.g. “*In your opinion*, *which would your colleagues’ attitude be towards the introduction of a PRP system?”*). Responses were provided on a scale from 1 (completely disagree) to 7 (completely agree).

**Fig 1:**
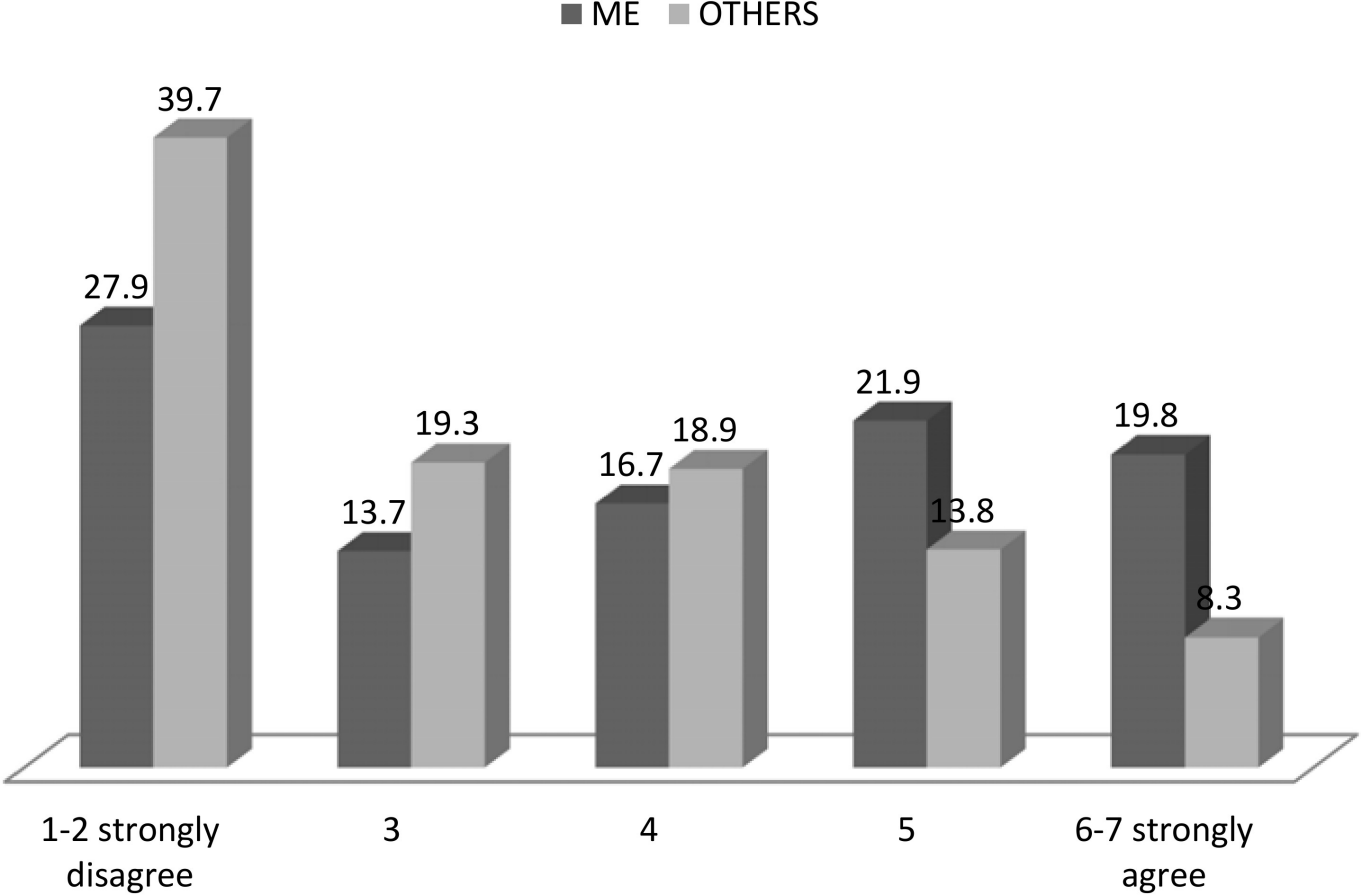
Own attitudes and beliefs regarding peers’ attitude towards PRP.

Fig 1 shows that approximately 20% of workers believe in a very successful implementation of the PRP system in their division. Interestingly, the perception the workers have on their coworkers’ reaction to the likelihood of a PRP system would be different. In terms of coworkers’ expectations regarding PRP implementation only 8,3% of workers believe that others’ attitude towards a PRP system could be positive. On the other hand, for those who believe that a PRP system could be unsuccessfully implemented, 39,7% believe that this would arise due to their coworkers’ reaction and only 27,9% due to their own.

Additionally, in Table 3, we present descriptive statistics of “own perception”, “my perception on others’ perceptions” and “me-vs-others misperceptions”. Misperceptions of others’ attitudes are calculated by subtracting the median of the behavior for each employee group from each respondent’s reported perception of their employee’s behavior. Therefore, each respondent has a misperception score which, if positive, indicated that they thought that other employees would be more negative towards the implementation of a PRP system in their job than their own attitudes. On average employees underestimate peers’ reaction on the successful implementation of the PRP system with an own mean of 3.86 versus a mean of 3.17 for others. To test whether there were significant misperceptions across the sample we conducted one-sample t-tests comparing the mean misperception for each category with a value of zero. All misperceptions were significantly different from zero.

**Table 3:** Mean ‘own perception’, ‘others perceptions’ and ‘me vs others misperceptions’.

	N Obs.	Own perception		Others perception		Misperception		t-test
		Mean	S.D	Mean	S.D	Mean	S.D	
All	567	3.86	1.85	3.17	1.60	0.82	1.60	12.17***
Clerks	338	3.76	1.87	3.08	1.59	0.91	1.59	10.55***
Managers	229	4.00	1.81	3.31	1.62	0.68	1.62	6.32***

*, **, *** indicate significant improvement at 10, 5, 1 percent levels respectively.

### 3. Empirical methodology

This study followed the methodology for measuring self-vs.-other discrepancies between attitudes by asking employees to express an opinion on what others *would think* regarding a possible implementation of a PRP scheme in their job. The methodology employed is based on regressions that relate the acceptability of PRP system to a set of attitudinal statements. The definition variables included in the model are presented in Table 1. The dependent variable is an ordered categorical variable taking on a finite number of outcomes, on a scale of 1 to 7, representing the lowest and highest rating respectively. (*“In your opinion*, *could an introduction of a PRP system be effectively implemented in your job (section)?”)*, which means that respondents can only express their responses on a non-linear scale. Therefore, the researcher does not know the respondent’s exact feeling but only the interval to which she/he belongs and for this reason, the respondent’s attitude is assumed to be a latent variable that is not directly observable and thus, an ordered probit model is applied.

In an ordered probit model, the latent probability of reporting a level of successfully implementation of a PRP system P^*^ is: *Own perception of the effective implementation of a PRP system*: *P*_*i*_ = Φ^−1^(*P*_i_^*^) = *ϑX*_*i*_ + *δS*_*i*_ + *ε*_*i*_ *e*_*i*_|*X* ∼ *Normal*(0,1) (Model 1), where *P*_*i*_ is the own perceived implementation of the PRP system in the public sector stated by individual *i*. *X*_*i*_ are the 11 attitudinal variables. *S*_*i*_ is a vector of control variables and *ε*_*i*_ is the error term. Assuming that *μ*_1_ < *μ*_2_ < … < *μ*_*j*_ where *μ*_1_,…*μ*_*j*_ are the cutoff points for the latent variable

$${\text{P}}^{*}=\{\begin{array}{l} 0,\;\text{if}\;\text{P}*\leq \mu_{1}\\ 1,\;\text{if}\;\mu_{1}\leq \text{P}*\leq \mu_{2}\\ \ldots \\ \text{j},\;\text{if}\;\text{P}*>\mu_{\text{j}} \end{array}$$

the parameters *θ*,*δ* and *μ* can be estimated by maximum likelihood.

In our model, we transform the five-point scale of the 11 attitudinal variables to binary variables. Therefore, we create 11 dummy variables equal to 1 if the individuals reported the highest score in the five-point scale and 0 otherwise.

The regression was re-run for the coworkers’ perception to identify differences in the explanatory factors of own and others’ perceptions using an ordered probit model. The dependent variable is again an ordered categorical variable based on the following question *“In your opinion*, *which would your colleagues’ attitude be towards the introduction of a PRP system?”* range from 1 (completely disagree) to 7 (completely agree).

A second model is specified to study *Individual perceptions on others beliefs of the effective implementation of a PRP system*: *L*_*i*_ = *αX*_*i*_ + *λS*_*i*_ + *u*_*i*_ (Model 2), where *L*_*i*_ is individual *i* perception over the others’ perceptions for the successful implementation of the PRP system in the public sector. *X*_*i*_ are the 11 attitudinal variables. *S*_*i*_ is a vector of control variables and *u*_*i*_ is the error term. The parameters *α*,*λ* and *μ* (the cutoff points for the latent variable) will be estimated by maximum likelihood. The results of the regressions are presented in Tables 4, 5 and 6.

**Table 4:** Ordered probit regression results of the categorical responses of “Own perception/own beliefs” versus “my perception on others’ perceptions/second order beliefs” of the effective implementation of a PRP system and “misperceptions”.

VARIABLES	Own beliefs	Second order beliefs	Misperceptions
			(zscore)
	(1)	(2)	(3)
	Coef.	Coef.	Coef.
A PRP system help the workforce to improve its productivity	0.292**	0.270**	-0.275**
A PRP system help public servants to better understand the organization values and priorities	0.678***	0.246	-0.234
A PRP systems in public administration discourage low-skilled applicants	-0.168	-0.254	0.215
A PRP system prompt employees to be interested in tasks related to financial incentives	0.0416	-0.394***	0.335***
A PRP system lead a public servant to an unethical behavior	-0.359**	-0.224	0.191
A PRP systems demotivate public servants that are intrinsically stimulated	-0.457**	-0.374*	0.268
A PRP system influence positively: supervisor-employee relationship	-0.0121	-0.160	0.105
A PRP system influence positively: relationships with colleagues	0.328	-0.0111	0.061
A PRP system influence positively: total pay	0.555***	0.396***	-0.352***
A PRP system infl. positive. job security feel	0.135	-0.136	0.091
A PRP system infl. positive. tensions in work	0.423***	0.188	-0.162
Job satisfaction	0.156*	0.302***	—0.251***
Male	0.171***	0.130***	-0.113**-
Constant			0.172**
Constant cut1	-0.806	-0.828	
Constant cut2	-0.285	-0.0881	
Constant cut3	0.112	0.414	
Constant cut4	0.586	1.010	
Constant cut5	1.347	1.662	
Constant cut6	1.912	2.157	
Log-likelihood	-933.7	-901.4	
R^2^			0.11
Observations	520	520	520

*, **, *** indicate significant improvement at 10, 5, 1 percent levels respectively.

**Table 5:** Summary statistics of the attitudinal traits towards the introduction of PRP scheme disaggregated by job roles.

Attitudinal traits		Mean (range 1–5)	Std.Dev.	t-stat.
Q 1. In your opinion, to what extend does a PRP system in public service help the workforce to improve its productivity?	Managers	3.70	1.13	1.61*
	Clerks	3.54	1.10	
Q.2. In your opinion, to what extent does a PRP system help public servants to better understand the organization values and priorities?	Managers	3.25	1.22	1.12
	Clerks	3.14	1.15	
Q.3. In your opinion, to what extent do PRP systems in public administration discourage low-skilled employees (applicants)?	Managers	2.98	1.29	2.75*
	Clerks	2.80	1.24	
Q.4. In your opinion, to what extent do PRP systems prompt public servants to be interested solely in tasks that are directly related to financial incentives?	Managers	3.81	0.93	0.64
	Clerks	3.76	0.92	
Q.5 In your opinion, to what extent may a PRP system lead a public servant to an unethical behavior?	Managers	2.99	1.21	1.19
	Clerks	3.11	1.18	
Q.6. In your opinion, to what extent may PRP systems demotivate public servants that are intrinsically stimulated?	Managers	2.54	1.23	3.28***
	Clerks	2.89	1.23	
Q.7. In your opinion, in which way could an introduction of a PRP system influence: supervisor-employee relationship (from 1 negative to 5 positive)	Managers	3.04	1.18	1.42
	Clerks	2.90	1.14	
Q.8 In your opinion, in which way could an introduction of a PRP system influence: relationships with colleagues (from 1 negative to 5 positive)	Managers	2.56	1.07	2.04**
	Clerks	2.37	1.08	
Q.9. In your opinion, in which way could an introduction of a PRP system influence: total pay (from 1 negative to 5 positive)	Managers	3.90	1.03	1.78*
	Clerks	3.74	0.99	
Q.10. In your opinion, in which way could an introduction of a PRP system influence: sense of job security (from 1 negative to 5 positive)	Managers	2.84	1.15	0.38
	Clerks	2.80	1.22	
Q.11. In your opinion, in which way could an introduction of a PRP system influence: tensions in work (from 1 negative to 5 positive)	Managers	3.19	1.30	2.26***
	Clerks	2.93	1.28	
Dependent variables				
In your opinion, could an introduction of a PRP system be effectively implemented in your job (section)?	Managers	4.00	1.81	1.48
	Clerks	3.76	1.87	
In your opinion, how could be your colleagues ‘reaction to the introduction of a PRP system	Managers	3.31	1.62	1.69**
	Clerks	3.08	1.59	
Control variable				
Your Job satisfaction from 1 to 7	Managers	4.48	1.35	1.03
	Clerks	4.36	1.43	

*** p<0.01

** p<0.05

* p<0.1

**Table 6:** Ordered probit regression results of the categorical responses of “Own perception” versus “my perception on others’ perceptions/second order beliefs” of the effective implementation of a PRP system split by employment position.

VARIABLES	Clerks_Own beliefs	Clerks_Second order beliefs	Managers_Own beliefs	Managers_Second order beliefs
	(1)	(2)	(3)	(4)
	Coef.	Coef.	Coef.	Coef.
A PRP system help the workforce to improve its productivity	0.364**	0.154	0.259	0.444**
A PRP system help public servants to better understand the organization values and priorities	0.586**	0.452*	0.895***	0.068
A PRP systems in public administration discourage low-skilled applicants	0.175	-0.242	-0.525**	-0.245
A PRP system prompt employees to be interested in tasks related to financial incentives	-0.008	-0.533***	0.148	-0.194
A PRP system lead a public servant to an unethical behavior	-0.298	-0.204	-0.456*	-0.177
A PRP systems demotivate public servants that are intrinsically stimulated	-0.554**	-0.185	-0.418	-0.872**
A PRP system influence positively: supervisor-employee relationship	-0.237	-0.173	0.187	-0.093
A PRP system influence positively: relationships with colleagues	0.349	-0.130	0.574	0.125
A PRP system influence positively: total pay	0.666***	0.466***	0.374**	0.280
A PRP system influence positively: sense of job security	-0.014	0.029	0.0996	-0.404
A PRP system influence positively: tensions in work	0.730***	0.097	0.185	0.114
Male	0.163	0.255**	0.164	0.336**
Job satisfaction	0.242***	0.118*	0.0881	0.151*
Constant cut1	-0.832	-0.816	-0.790	-0.875
Constant cut2	-0.177	-0.0673	-0.470	-0.144
Constant cut3	0.162	0.403	0.0207	0.412
Constant cut4	0.640	1.033	0.507	0.963
Constant cut5	1.399	1.722	1.312	1.602
Constant cut6	1.852	2.197	2.069	2.135
Log-Likelihood	-540.91	-519.93	-373.79	-373.44
Observations	307	307	213	213

*, **, *** indicate significant improvement at 10%, 5%, and 1% levels, respectively.

## 4. Results

This section presents: a) the results of the employee’ perception about the successful implementation of a PRP system in the public sector (Table 4, column 1), b) the estimation results for perceptions on others’ attitudes (Table 4, column 2) and the misperceptions defined as “me vs others” discrepancies (Table 4, column 3). More specifically, the dependent variable misperception is an ordered categorical variable which also takes negative values. We define this variable as z-scores measuring the number of standard deviations between a given response and the mean. According to Freeman (1978) this unit transformation preserves the rank-order of the values and yields results that are qualitatively similar to those that the original variables would have yielded. c) the estimation results disaggregated by job position (Table 5, Table 6, column, 1–4).

### 4.1. Are there differences between “my beliefs” and “my beliefs on others’ beliefs-norm”?

In general, respondents perceived their colleagues’ attitudes towards a successful implementation of a PRP system to be less positive than their own self-reported attitudes. More specifically, the individuals who strongly agree that “a PRP system will help public servants to better understand the organization values and priorities” and “will create more tensions in work” are more likely to believe that a PRP system could be effectively implemented in their section (own perception) whereas it has no impact on their beliefs concerning their colleagues’ attitude towards the introduction of a PRP system (norm). In addition, workers who strongly agree that such “a reward system could not lead a public servant to an unethical behavior” are less likely to believe that a PRP system could be effectively implemented in their section (own perception) whereas it has no impact on their beliefs concerning the norm.

### 4.2. Are there similarities between “my beliefs” and “my beliefs on others’ beliefs”?

There are also some similarities of perceptions observed across the “me vs others” dimension. For example, employees who strongly agree that a PRP system will help the workforce to improve its productivity are more likely to believe both for themselves and for their coworkers that a PRP system could be effectively implemented. Probably in employees’ perception, performance improvements coincide with improvements in management practices, e.g. clearly defined priorities and values. However this is in contrast with Weibel et al. [16] and Schmidt et al. [10] who find that the effect on employee performance is less clear and could even be negative.

Job tasks and responsibilities are usually complex in public administration. This so-called ‘multitasking problem’ [16] creates additional difficulties in the precise measurement of performance. Performance appraisal systems have been rather problematic in public organizations. The existence of quotas in extrinsic rewards and favoritism create feelings of unfairness among employees [10, 19, 20]. Public sector employees are more risk averse and intrinsically motivated than those of the private sector [19, 16]. Thus, PRP may affect their efforts negatively. In line with the above literature, this study shows that those who strongly agree that PRP system could demotivate public servants that are intrinsically stimulated are less likely to believe to a successful implementation of a PRP system.

Studies on PRP show that constraints in funding of public sector organizations restrict the level of monetary rewards. As a consequence, the effect of PRP is rather limited, since employees may not value low monetary rewards [16, 19, 21]. This study does not confirm these findings. We show that the wage positively influences the expectation of a successful implementation of PRP schemes.

Evidence also shows that the application of PRP in the public sector undermines employee morale and teamwork [19, 22]. The notions of profit and loss are rather specific to the private sector, and may be unfamiliar to civil servants [17, 23]. Our study shows that there will be no significant effect on a worker’s relationship with a supervisor and with colleagues.

Additionally, those who are more satisfied with their job are more positive to an introduction of a PRP system. Job satisfaction, which would include all monetary and especially non-monetary features such as the amount of discretion that is exercised, how challenging or stimulating the job is, the degree of skill utilization etc., suggests that workers have a preference for financial rewards based on performance. More satisfied workers are likely to be more productive [24], hence affecting their likelihood of receiving PRP in the future [25]. Males are more positive towards PRP wage systems.

In order to further investigate the misperceptions between “me vs others”, we run a linear regression using as dependent variable the misperception score. As stated earlier, misperceptions of others’ attitudes are calculated by subtracting the median of the behavior for each employee group from each respondent’s reported perception of their peers’ behavior. Since the dependent variable is not naturally ordinal, and takes negative values it was transformed into z-scores measuring the number of standard deviations between a given response and the mean. According to Freeman [26], this unit transformation preserves the rank-order of the values and yield results that are qualitatively similar to those the original variables would have yielded The coefficients of “improve productivity” and “total wage” which are significant and negative indicate that individuals tend to be less optimistic than their beliefs concerning their colleagues attitude would be towards the successful implementation of a PRP system. The coefficient “A PRP system prompts employees to be interested in tasks related to financial incentives” is significant and positive indicating that they thought that other employees would be more negative towards the implementation of a PRP system in their job than they would be themselves.

### 4.3. Clerk- manager differences

Research suggests that work roles influence perception and have an influence on behavior of work aspects. Humphrey [6] finds that subordinate workers undervalue their fellow subordinates and overevaluate their managers concluding that organizational factors systematically bias the information that actors have about each other. Previous literature on organizational behavior has shown that organizational factors may distort workers’ perceptions of each other revealing that the clerks rate managers higher on leadership, intelligence and other important role-related traits than the fellow clerks [6]. The above literature implies that since managers are higher in the organizational hierarchy and perceive that they do more responsible work, they will probably view the introduction of PRP system more favorably than clerks.

Therefore, we conduct our research on job roles in order to highlight whether there are job role differences in terms of the perceived implementation of the PRP system. We split the sample into two groups, those with a clerical position and those with a managerial position. According to Table 3, on average, clerks perceived their peers’ attitudes to be less positive than they actually were with an own mean of 3.76 versus a mean of 3.08 of others. The misperception score on clerks’ group (0.91) is significantly higher than the 0.68 mean misperception of managers, based on an one-sided t-test. The result also holds allowing for unequal variances.

Table 5 presents summary statistics of the responses. The average numerical answers of 5 out of 11 attitudinal traits from both managers’ and clerks’ responses are greater than the midpoint of the scales (3). The *t-statistics* for these job position differences indicate high statistical significance for 6 out of 11 attitudinal traits. Managers are more optimistic than clerks that a PRP system will help public servants to better understand the organization values and priorities (Q.2), establishing an organizational culture oriented towards productivity (Q.1), discouraging low-skilled applicants (Q.3), and positively influencing total pay (Q.9). On the other hand, clerks believe more than managers that a PRP system will influence negatively the relationship with their colleagues (Q.8), demotivate public servants that are intrinsically stimulated (Q.6), whereas it will produce fewer tensions in work (Q.11). Summing up, we show that managers tend to be more optimistic than clerks regarding the successful implementation of PRP systems. Therefore, managers have a stronger belief in the positive aspects of PRP systems, whereas clerks hold stronger beliefs regarding their negative aspects.

The purpose of the following section is to highlight whether there are manager-clerk differences using econometric models (both within their fellows and between their job positions) in terms of their own beliefs and their beliefs on others’ beliefs towards the successful implementation of PRP system in their job.

According to Holmstrom [27], the underlying premise behind all such schemes is that workers are motivated primarily by monetary rewards and that an increase in remuneration based on performance will invariably lead to an improvement in performance along the lines desired by the management. In this study we find that all workers believe that pay, per se, is not necessarily a potential motivator and employees would not be interested solely in tasks related to financial incentives. However, clerks who strongly agree with the statement that a PRP system prompt employees to be interested in tasks related to financial incentives are less likely to believe for others (norm) towards a successful implementation of a PRP system however it has no impact for their own beliefs.

For clerks, who strongly agree that a PRP system will help public servants to improve their productivity are more likely to believe in a successful implementation of a PRP scheme, whereas for managers this statement has no impact for their own beliefs and has positive impact for others’ beliefs.

Many workers could probably become very concerned about their level of job security. In a survey of US workers both in the public and private sectors, for example, found that many workers were very concerned about their level of job security [28]. However this study, in line with Pouliakas & Theodossiou [29] finds that contingent rewards do not affect job security. This effect could arise because public sector jobs in Greece still provide long term employment stability.

In pay-for-performance schemes managers can exactly set the values and priorities that employees must achieve and this helps managers to focus on what is central to their operation. We found that clerks who strongly believe for themselves and for their coworkers that a PRP can indeed help them understand the desired outputs consider in a successful implementation of a PRP system in their section. Finally, it is found that managers who strongly believe that pay-for-performance schemes could discourage and lead public servants to an unethical behavior are more negative to an introduction of a PRP system effectively. Whereas for clerks this statement has no impact. This could probably be explained by the fact that PRP schemes give managers more flexibility to reward the best workers, but also provide systems to stigmatize the least effective ones.

Overall the results of this study reveal that both managers and clerks perceive differently on selected attitudinal traits and hold heterogeneous beliefs on others’ expectations regarding a successful implementation of PRP.

## 5. Conclusions

This paper investigates how public sector employees perceive the forthcoming introduction of a PPR system making a distinction between individual perceptions and perceptions on others’ attitudes. It is the first of its kind in the country, since PRP schemes have never been used in the Greek public sector before. Although most studies focus on the impact of PRP on employee motivation and organizational outcomes long *after* the adoption of PRP, this study is conducted *prior* to its introduction. It is a quantitative attempt aimed at exploring in depth employees’ perceptions on the initiation of PRP schemes in a public organization. As Schmidt et al. [10] point out, the acceptance of a PRP system is more likely if employees are involved in the design of this system and understanding their preferences is a necessary first step.

Contrary to what one would have thought, public sector employees in Greece are far from unanimously negative regarding the adoption of performance contingent pay. However, they seem to “delegate” the opposition to others around them, in an apparent effort to signal a pro-efficiency own attitude but put the blame for a more conservative view on others. This could be explained as the wish to comply with some objectively correct measure, which would face objections by others.

The explanatory factors of own attitudes towards performance-contingent pay are similar to those of beliefs on others’ attitudes on the following points: a PRP system will help the workforce to improve its productivity, it will have a positive effect on wage, but it could demotivate public employees that are intrinsically stimulated.

Moreover, workers have heterogeneous attitudes and hold heterogeneous beliefs on others’ expectations regarding the fact that a PRP system will help public employees to better understand the organization values and priorities, and that after the implementation of such schemes public servants will be interested solely in tasks that are directly related to financial incentives.

Social norms play a central role in the formation of individual beliefs and behavior and the differences in how you perceive yourself from how you expect others to think may provide important information on the tension between the true individual attitudes and a normative representation of one’s own preferences. The understanding of this process creates opportunities for eliminating misperceptions and successfully implementing reforms in public administration in the near future. The results reported here may be valuable to policy makers and human resources managers, helping scholars to understand employees’ views, concerns or even fears, and suggest ways of better initiating a PPR scheme. Relevant studies have been largely quantitative, paying insufficient attention to employee idiosyncratic views with respect to PRP systems [22, 19]. Practitioners may incorporate employees’ perceptions, social perceptions, opinions and concerns in order to create an effective PRP system.

## Supporting information

S1 File STATA Data file. (DTA)
